# Overexpression of melanoma inhibitory activity (MIA) enhances extravasation and metastasis of A-mel 3 melanoma cells in vivo

**DOI:** 10.1054/bjoc.2000.1424

**Published:** 2000-11

**Authors:** M Guba, A-K Bosserhoff, M Steinbauer, C Abels, M Anthuber, R Buettner, K-W Jauch

**Affiliations:** Department of Surgery, University of Regensburg; Institute of Pathology; Department of Dermatology, RWTH Aachen Medical School; University of Regensburg, Germany

**Keywords:** MIA, intravital microscopy, tumour cell invasion, melanoma

## Abstract

The secreted MIA protein is strongly expressed by advanced primary and metastatic melanomas but not in normal melanocytes. Previous studies have shown that MIA serum levels correlate with clinical tumour progression in melanoma patients. To provide direct evidence that MIA plays a role in metastasis of malignant melanomas, A-mel 3 hamster melanoma cells were transfected with sense- and antisense rhMIA cDNA and analysed subsequently for changes in their tumorigenic and metastatic potential. Enforced expression of MIA in A-mel 3 cells significantly increased their metastatic potential without affecting primary tumour growth, cell proliferation or apoptosis rate in hamsters, compared with control or antisense transfected cells. Additionally, MIA overexpressing transfectants showed a higher rate of both tumour cell invasion and extravasation. Cells transfected with MIA antisense generally exerted an opposite response. The above changes in function attributed to the expression of MIA may underlie the contribution of MIA to the malignant phenotype. © 2000 Cancer Research Campaign


*British Journal of Cancer* (2000) **83**(9), 1216–1222
© 2000 Cancer Research Campaign
doi: 10.1054/ bjoc.2000.1424, available online at http://www.idealibrary.com on IDEAL®

### Overexpression of melanoma inhibitory activity (MIA) enhances extravasation and metastasis of A-mel 3 melanoma cells in vivo

**M Guba[1*], A-K Bosserhoff[2,3*], M Steinbauer[1], C Abels[3], M Anthuber[1], R Buettner[2] and K-W Jauch[1]**

Department of Surgery[1], University of Regensburg; Institute of Pathology[2], RWTH Aachen Medical School; Department of Dermatology[3], University of Regensburg, Germany

**Summary** The secreted MIA protein is strongly expressed by advanced primary and metastatic melanomas but not in normal melanocytes. Previous studies have shown that MIA serum levels correlate with clinical tumour progression in melanoma patients. To provide direct evidence that MIA plays a role in metastasis of malignant melanomas, A-mel 3 hamster melanoma cells were transfected with sense- and antisense rhMIA cDNA and analysed subsequently for changes in their tumorigenic and metastatic potential. Enforced expression of MIA in A-mel 3 cells significantly increased their metastatic potential without affecting primary tumour growth, cell proliferation or apoptosis rate in hamsters, compared with control or antisense transfected cells. Additionally, MIA overexpressing transfectants showed a higher rate of both tumour cell invasion and extravasation. Cells transfected with MIA antisense generally exerted an opposite response. The above changes in function attributed to the expression of MIA may underlie the contribution of MIA to the malignant phenotype. © 2000 Cancer Research Campaign

**Keywords**: MIA; intravital microscopy; tumour cell invasion; melanoma

Despite the fact that worldwide incidence of melanoma is increasing more than in any other neoplastic disease, the molecular basis of malignant melanoma progression has remained largely unknown (Kopf et al, 1995). Changes associated with the transition of melanoma cells from radial growth phase to vertical growth phase are, as yet, not well defined. Since metastasis depends on the completion of a multistep process involving the survival and growth of a unique subpopulation of cells with metastatic properties, more information is obviously needed regarding the genetic changes underlying melanoma tumorigenesis and progression to provide further insight into the development of this form of cancer (Stetler Stevenson et al, 1993 Fidler, 1996).

What is of particular relevance to this paper are recent results with a novel secreted protein MIA in melanoma.

MIA has been previously identified within growth-inhibitory activities purified from tissue culture supernatant of malignant melanoma cells in vitro (Apfel et al, 1992). A single copy gene encoding for a secreted 11 kDa protein was cloned and allocated to human chromosome 19q13.32–q13.33 (Blesch et al, 1994; Koehler et al, 1996). MIA expression is in some physiological conditions associated with embryonic development and intense tissue remodelling where MIA mRNA was identified independently by a differential display approach comparing differentiated and dedifferentiated cartilage cells in vitro (Bosserhoff et al, 1997b). In adult tissues MIA was mainly detected in cartilage and malignant melanoma. Comparing the expression in normal skin, melanocytes, melanocytic naevi and malignant melanomas MIA mRNA levels were observed to parallel progressive malignancy of melanocytic tumours. Previous studies have shown that MIA protein serum levels correlate with the clinical tumour stage in melanoma patients (Bosserhoff et al, 1997a, 1998).

These observations raise the question as to whether malignant transformation of melanocytes may be associated with changes in the expression of MIA protein. We hypothesized that overexpression of MIA is likely to be linked to melanoma progression by direct or indirect influence on growth, invasion, and metastasis of the tumour. As a first step in this line of investigation, we report on the functional consequences of MIA expression in a hamster melanoma cell line. The results obtained indicate a direct correltion between MIA expression and metastatic capacity in melanoma cells. Furthermore, we identified both enhanced invasion and extravasation of tumour cells as a specific step in MIA function critical for the metastatic process.

### MATERIAL AND METHODS

### Cell culture

A Syrian Golden hamster derived (Dellian et al, 1996) amelanotic melanoma cell line, A-mel 3 (a kind gift from K Messmer, University of Munich) was grown in RPMI supplemented with 10% fetal calf serum (FCS), 2 mM of L-glutamine, 100 units/ml penicillin and 0.1 mg/ml streptomycin. Cultures were maintained in a humidified 5% $CO_2$ atmosphere at 37°C. After transfection with sense or antisense MIA constructs, the cells were selected and

*Received 24 March 2000*
*Revised 23 June 2000*
*Accepted 28 June 2000*

*Correspondence to*: M Guba

*Both authors have contributed equally to this work maintained with 50 µg/ml G418 (Sigma Aldrich Deisenhofen, Germany) in supplemented RPMI medium.

### Stable transfection of melanoma cells with sense and antisense MIA

A panel of A-mel 3 cell clones varying with respect to MIA expression were established by stable transfection with human MIA-sense or antisense expression plasmids, controlled by a CMV-promoter (Blesch et al, 1994) and cotransfected with pCDNA3 (Invitrogen NV Leek, Holland), containing the selectable marker for neomycin resistance. Controls received pCDNA3 alone. Transfection was performed using lipofectamin (Gibco-BRL, Eggenstein, Germany) according to the manufacturer's instructions. One day following transfection, cells were placed into a selection medium containing 50 µg/ml G418 (Sigma Aldrich Deisenhofen, Germany). After 25 days of selection, individual G418-resistant colonies were subcloned. MIA expression levels of these clones were determined by a quantitative MIA specific ELISA (Boehringer Roche Mannheim, Germany) and Western blot analysis of 24 h tissue culture supernatants.

### Tumorigenicity and organ colonization assays

Cultured cells were harvested by brief trypsin-EDTA (Gibco-BRL) treatment, resuspended in serum-free RPMI. $1 \times 10^6$ cells in a total volume of 0.2 ml were injected subcutaneously into the dorsal skin-fold of hamsters. The tumour size was measured three times a week with a caliper and the palpable inguinal and axillar lymph nodes were assessed. Tumour volume was determined using the formula (tumour volume = $0.863 \times l \times w \times h$) as described previously (Dellian et al, 1996). The incidence of lymphatic dissemination was estimated by a score system, whereby palpable axillar and inguinal lymph nodes, received one point each, leading to a maximal score of 4 points/hamster ($n$ = 11/group). Lung colonization was determined after 35 days by direct visualization of surface colonies under a dissecting microscope ($n$ = 11/group).

Post-mortem examinations were conducted on all animals. Tumour tissues, lymph nodes and lungs were fixed in 10% PBS-buffered formalin, embedded in paraffin and sections were stained with haematoxylin (Merck Darmstadt, Germany) & eosin or by TUNEL (ApopTag™ Oncor, Appligene, Heidelberg, Germany) for histological examination. Frequency of apoptosis is indicated as the mean ± SEM of 8 high power fields observed per primary tumour.

### In vitro growth assays

In vitro anchorage-dependent proliferation of variant A-mel 3 subclones was assessed as previously described (Buettner et al, 1991). Briefly, cells were plated in triplicate into 24-well microtitre plates ($2 \times 10^7$ cells/well) and the effect of high (10%) and low (1%) concentrations of FCS was determined at 20, 50, 75 and 100 h. Proliferation of the differently transfected A-mel 3 melanoma cells was determined by direct cell counts.

### In vitro invasion assay

In order to investigate the effects of MIA expression on the ability of A-mel 3 cells to migrate through a filter or to invade a biological barrier a modified Boyden chamber was prepared as previously described (Jacob et al, 1995). Briefly, a polycarbonate filter (Nucleopore Coming Costar, Heidelberg) with 8 µM pores coated with matrigel (Becton Dickinson, Heidelberg) was used as a barrier. The lower compartment was filled with fibroblast-conditioned medium as a chemoattractant. A-mel 3 clones ($2 \times 10^5$ cells/well) were placed in the upper compartment of the chamber in RPMI medium (without FCS) and incubated at 37°C in a humidified 5% $CO_2$ incubator. After 24 hours, the upper surface of the inserts was wiped with cotton swabs, and the inserts were stained with haematoxylin and eosin (H & E). Each experiment was performed twice with each sample as triplicate. Cells that had migrated through Matrigel and filter pores to the lower surface were fixed, stained with H & E and 15 random fields were counted at 200-fold magnification. The invasion capacity was expressed as the number of cells that migrated per high-power field.

### Intravital video microscopy (IVM)

IVM was performed using a modified technique as previously described by Morris et al. (1993) Briefly, cells were labelled with either Calcein-AM (Sigma Aldrich) for short term observation (0–2 h) or fluorescent microspheres (Fluoresbright Carboxylated Microspheres, Molecular Probes, Eugene, OR, USA) (24 h) and $10^6$ cells in a total volume of 0.5 ml were injected into a mesenteric vein of 40–60 g male Syrian Golden hamsters. Analysis by IVM was performed 0–2 h or 24 h after cell injection (magnification: × 20 and × 40). During the whole procedure hamsters were anaesthetized with urethane (10 mg/100 g b/w) and placed on a heated custom-made platform. The left liver lobe was exteriorized through an abdominal midline incision, immobilized on a specially designed stage and covered with a thin cover slip. Hepatocytes were fluorescently labelled with intravenously injected sodium fluorescin (2.0 µmol/kg b/w), creating a negative stain of the liver sinusoids. In vivo microscopy was performed using a Zeiss fluorescence microscope in epilumination (Axioplan objectives × 20/0.40 korr, Zeiss, total magnification × 460). Images were recorded through a CCD video camera attached to the microscope and a S-VHS video recorder to enable off-line analysis. Individual cells were positively identified by green fluorescence and classified as either intravasal, in the process of extravasation, or extravasated cells. Viability was confirmed by propidium iodide exclusion after each experiment.

### Liver colonization model

Variant A-mel 3 clones were prepared as single cell suspensions in serum free RPMI at a concentration of $2 \times 10^6$/ml. 0.5 ml ($1 \times 10^6$ cells, viability 97%) were injected into a branch of the superior mesenteric vein of the hamsters. Animals were sacrificed by a high dose of phenobarbital sodium (250 mg/kg i.p.) after 20 days. At that time control hamsters became moribund. Metastases on the liver surface were counted and measured using an ocular micrometer.

### RESULTS

### Transfectants

Based on the high conservation of the MIA sequence among different species, we expected functional properties of the MIA protein to be conserved (Bosserhoff et al, 1998). Most recently we were able to sequence the hamster MIA. Hamster MIA has only one amino acid substitution as compared to the human protein in a

© 2000 Cancer Research Campaign 

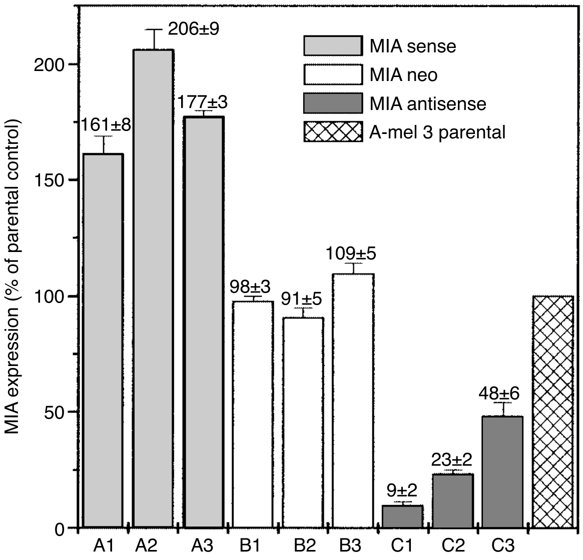

**Figure 1** MIA ELISA. MIA secretion of sense, antisense and neotransfected A-mel 3 cell clones in 24 h tissue culture supernatant were measured by ELISA. Significantly higher levels of total MIA expression were seen in MIA sense A-mel 3 clones (clones A1–3) as compared with the parental A-mel 3 cells. Although expression of the vector alone did not appear to change the levels of MIA expression in A-mel 3 cells (clones B1–3), a significant lower expression in cases where the cells had been transfected with the antisense constructs was observed (clones C1–3). For further analysis clone A2 (MIA sense), clone B1 (MIAneo) and clone C1 (MIA antisense) were chosen

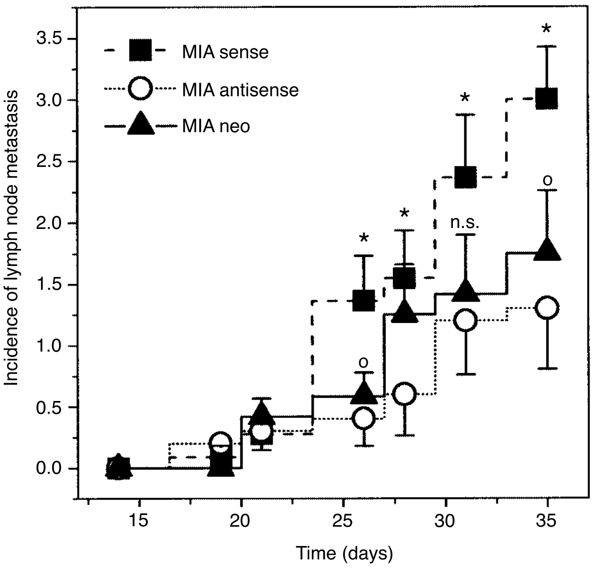

**Figure 2** Enhancement of lymph node metastasis development with high levels of MIA. A-mel 3 clones expressing high (MIA sense) or low levels (MIA antisense) of MIA and control cells (MIA neo) were injected subcutaneous in hamsters ($1 \times 10^6$ cells/animal). Metastasis was suspected by palpation of lymph nodes and positively diagnosed by the presence of palpable lymph nodes at any of the 4 typical sites, i.e. left and/or right in the axillar and/or inguinal region. For each histologically proven positive site one point was scored. Incidences per positive site are presented as the mean ± SEM; (**P* < 0.05 MIA sense vs. MIA antisense, °vs. MIA neo)

non-functional domain. Therefore, the human MIA should be fully functional in hamster cells. Levels of MIA expression in sense or antisense lipofectamin infected A-mel 3 melanoma cells were quantitatively examined by ELISA (Figure 1). Significantly higher levels of total MIA expression (human + endogenous hamster MIA) were seen in MIA sense A-mel 3 clones (clone A1–3) as compared to the parental A-mel 3 cells (endogenous hamster MIA only). Transfection of the vector alone did not appear to change the levels of MIA expression in A-mel 3 cells (clone B1–3). Cells transfected with the antisense constructs (clone C1–3) had significantly lower MIA expression levels.

The metastatic behaviour of three different subclones (1–3) derived from each sense-, antisense- and control-transfection procedure were studied in preliminary experiments to avoid artifacts due to clonal variance. For more detailed analysis clone A2 (MIA sense), clone B1 (MIA neo) and clone C1 (MIA antisense) were used (Figure 1). The other two clones of each kind (MIA-sense and -antisense) behaved similar to the one shown. Recombinant human MIA (rhMIA) expression was also confirmed by Western blot analysis where it migrated as a single band with a MW of approximately 11 kDa (data not shown).

### Tumorigenicity and metastatic potential

The metastatic potential of MIA sense and antisense transfected cells was determined in a spontaneous metastasis assay after subcutaneous implantation. To that end, hamsters were injected subcutaneously with $1 \times 10^6$ MIA sense, MIA antisense or MIA neo cells and with subsequent assessment of both development of the primary tumour and lymphatic metastases. All variant A-mel 3 clones (MIA sense, MIA antisense, MIA neo) grew in hamsters (100% tumour uptake) leading to visible tumours (tumour volume, 50–200 $mm^3$) within 3 days. After 5 weeks tumour volumes reached 2870–9320 $mm^3$ without any obvious correlation to their MIA expression levels. The first palpable lymph nodes appeared after two weeks, in the inguinal and later in the axillary region. After 5 weeks all hamsters bearing MIA sense tumours had developed intense lymphatic metastasis while lymph node metastases were rarely observed in animals with MIA antisense tumours. Thus, MIA expression levels correlated positively with the incidence of lymphatic metastases, but were independent of the volume of the corresponding primary tumour (Figure 2). In all autopsied hamsters both macroscopic and microscopic investigation of lymphatic nodes, lung, liver, spleen and brain did not reveal any trace of tumour metastases in organs other than the lymphatic nodes and the lung during the observation period of 35 days. At this time animals were sacrificed and the number of lung metastases were counted. As shown in Figure 3 the MIA sense clone produced a high number of lung tumour colonies (mean = 170) in all injected hamsters (incidence 11/11). In contrast, the MIA antisense transfected cells produced very few lung metastases (mean = 4) in individual animals (incidence 6/11). Although MIA overexpression led to a dramatic increase in the number of metastases, we found no proof for a correlation between primary tumour size and corresponding MIA-level. With A-mel 3 subclones, an overlapping range of metastatic tumour sizes was observed.

The ability to produce lymphatic or lung metastasis by transfected A-mel 3 cells was not due to differences in cell division time, as no significant differences in cell doubling times were found when cultured in vitro in the presence of high (10%) and low (1%) concentrations of FCS (Figure 4). In cultures containing 1% FCS (limiting conditions) all transfected cells grew more slowly however cell doubling times of MIA sense transfected cells were indistinguishable from the MIA antisense or MIA neo cells (30–34 h).

© 2000 Cancer Research Campaign

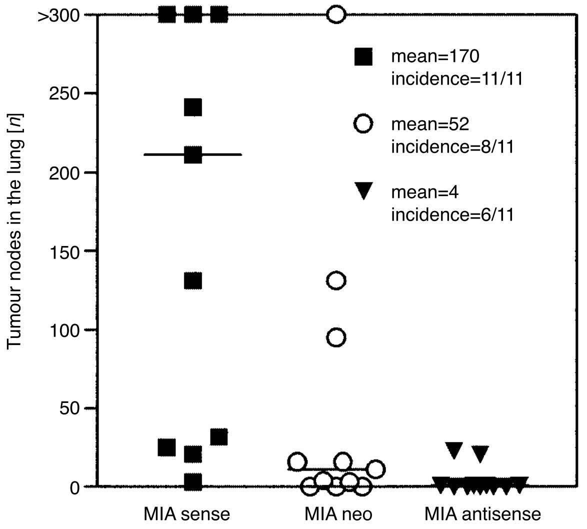

**Figure 3** MIA overexpression enhances spontaneous pulmonary metastasis. Five weeks following subcutaneous tumour implantation animals were sacrificed and lung metastases were estimated by assessing tumour nodules under a dissecting microscope. Lung metastasis was significantly enhanced by MIA overexpression, whereas lung metastasis was nearly abrogated in the MIA depleted subclone. Each point denotes a single value. A horizontal line gives the median value. The mean (M) and incidence (I) is indicated (**P* < 0.05 MIA sense, antisense vs. MIA neo)

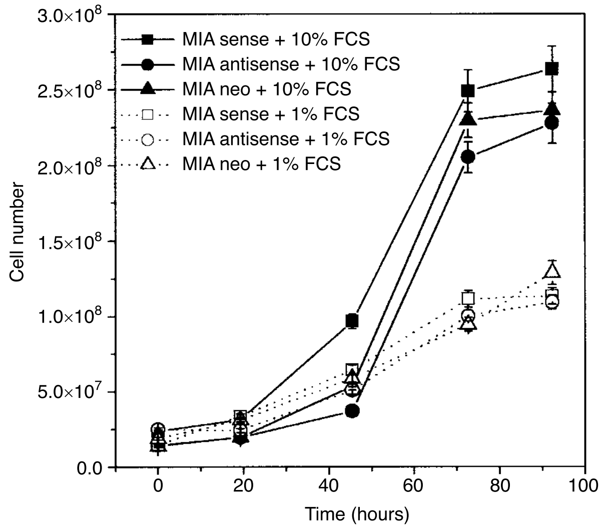

**Figure 4** In vitro proliferation characteristics of variant A-mel 3. Cells were trypsinized, washed, and $2 \times 10^7$ cells were plated in triplicate wells with either 1% or 10% FCS. Cell counts were performed using a haemocytometer. Results are presented as the mean ± SEM

To determine whether reduced expression of MIA affects development of metastases by triggering in vivo apoptosis, apoptotic cells in corresponding primary tumour sections were determined by terminal nick end-labelling (TUNEL) staining of DNA fragmentation. The frequency of apoptosis in MIA sense tumours (19 ± 6 cells/hpf) was not significantly different from those of MIA neo (17 ± 10 cells/hpf) or antisense tumours (19 ± 9 cell/hpf). Thus, the presence of high levels of MIA in the primary tumour appears to promote metastasis of A-mel 3 by mechanisms other than growth and apoptosis.

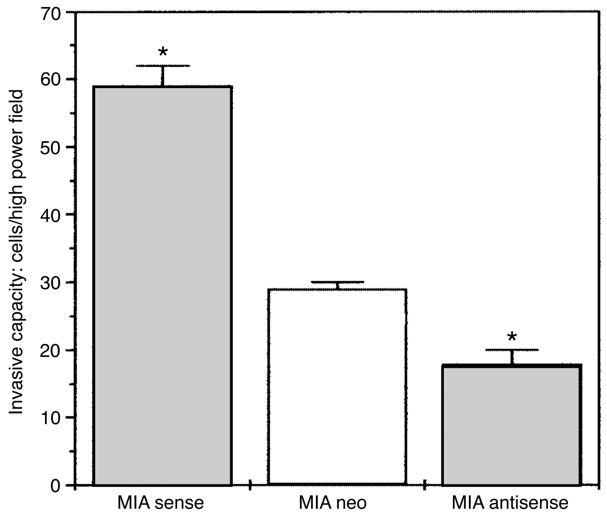

**Figure 5** Invasive potential of A-mel 3 transfectants. In vitro invasion capacity through Matrigel-coated filters. Fibroblast-conditioned medium was placed as a chemoattractant into the lower Boyden Chamber compartment. Invasive cells were counted in 15 random fields, as described in the Methods section. Each group comprised of 6 replicates in 2 independent experiments. Results are presented as the mean ± SEM (**P* < 0.05 MIA sense, antisense vs. MIA neo)

### Enhanced invasion and extravasation define the critical steps for MIA function in melanoma metastasis

To define specific steps in tumour metastasis where MIA function is critical, we then investigated the influence of MIA expression levels on the ability of the three clones to both migrate and invade in an in vitro system. Enhanced invasion capacity of tumour cells in vitro is generally associated with an aggressive behaviour in vivo. After 24 hours of culture, cells that had invaded through the basement membrane Matrigel and migrated through the pores to the other side of the filters were counted (Figure 5). The number of MIA sense cells that migrated within 24 h was drastically increased (59 ± 3 cell/hpf), as compared to control cells (29 ± 1 cell/hpf). MIA antisense transfected cells lead to a reduction of invasive and migratory properties (18 ± 2 cell/hpf). Overexpression of MIA caused an increase in invasion (203%) and MIA depletion a decrease (69%) relative to MIA neo cells. No significant morphological changes in either transfected cell line were observed.

To test these encouraging in vitro results in vivo, we then were interested in the metastatic behaviour of transfectants, especially in the step involving extravasation, which follows tumour cell passage through the blood stream and is thought to involve similar mechanisms. We therefore developed an in vivo model that allows us to study the temporal sequence of events occurring during the early steps of tumour cell implantation in host organs. Intravital microscopy enabled the identification of individual tumour cells following intraportal injection in the hepatic microcirculation and subsequent observation of the fate of these blood borne micrometastases.

Initial tumour cell arrest (during the first 2 h) was not affected by MIA expression. Contrary to circulating leucocytes, tumour cells showed no temporary or firm adhesion to the endothelial lining of large vessels. Tumour cells were arrested by size restriction in tapering sinusoids, consistent with plain mechanical arrest (Figure 7A). Within the initial observation period of 2 hours,

*© 2000 Cancer Research Campaign* 

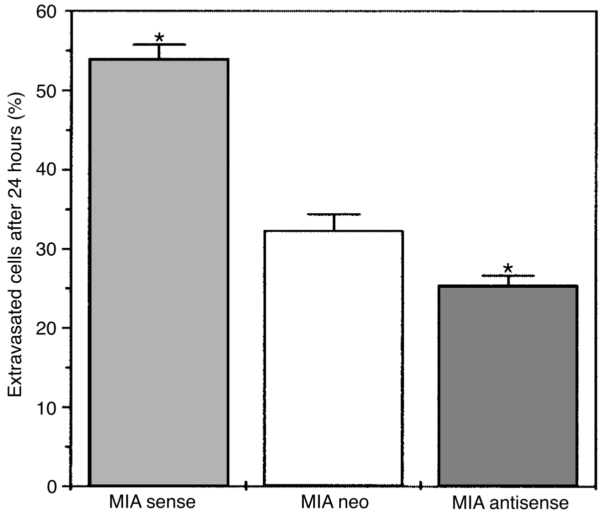

**Figure 6** Tumour cell extravasation in the microcirculation of the liver. The extent of extravasation in each hamster was enumerated as percentage of cells that had extravasated by 24 h after tumour cell injection. Cells were characterized as intravasal, in the process of extravasation or extravasated. Each observation involved at least 100 cells. Results presented as the mean ± SEM; $n = 6$ (*$P < 0.05$ MIA sense, antisense vs. MIA neo)

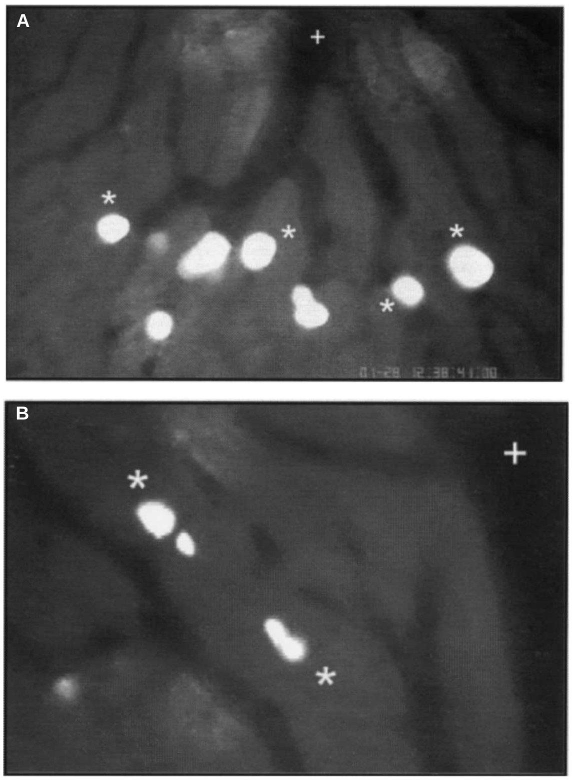

**Figure 7** Views of tumour cells by intravital microscopy. (**A**) Intravasal A-mel 3 MIA antisense melanoma cells (*) in the microcirculation of the liver 30 min after intraportal injection. Cells were labelled with fluorescent microspheres and were viewed by intravital microscopy using epifluorescence. Note, all cells were arrested by size restriction. (**B**) Extravasated A-mel 3 MIA sense cells (*) 24 hours after intraportal injection. A-mel 3 MIA sense cells extravasated into the hepatocyte layer from adjacent sinusoid (+)

lodged tumour cells gradually deformed, with no significant loss of viability (~95%) as determined by in vivo propidium iodide

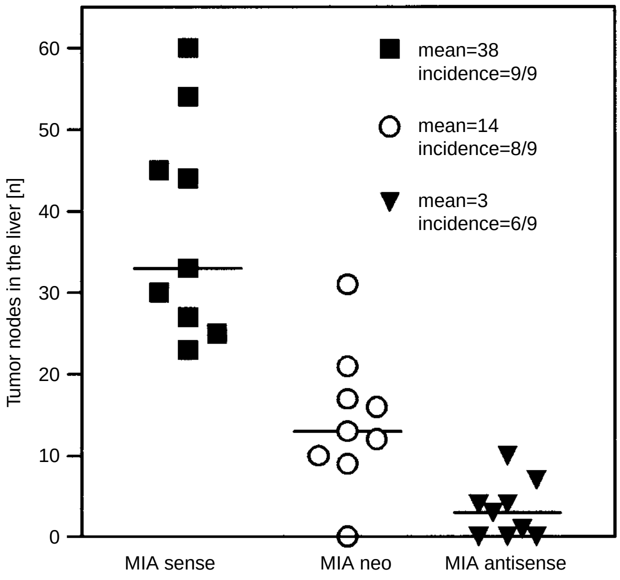

**Figure 8** Liver metastasis after intraportal injection of tumour cells. 20 days after intraportal inoculation of $10^6$ tumour cells, animals were sacrificed and liver metastasis was estimated by counting superficial tumour nodes using a dissecting microscope. Liver metastasis was significantly enhanced by MIA overexpression, and in parallel almost abrogated in the MIA depleted subclone. Each point denotes a single value. A horizontal line gives the median value. The mean (M) and incidence (I) is indicated (*$P < 0.05$ MIA sense, antisense vs. MIA neo)

exclusion. While these processes remained unaffected by MIA expression, the following extravasation process as previously described by Koop et al. (1995) was indeed affected by MIA. Extravasation was initialized by polarization of the tumour cell, formation of pseudopodia and retraction from the endothelial lining, followed by translocation into the liver parenchyma. After completion of the extravasation process tumour cells were completely localized between the liver cells (Figure 7B). Tumour cells that could not accomplish the extravasation process were gradually destroyed by haemodynamic force. The speed of extravasation positively correlated with MIA expression. After 24 hours significantly more (2-fold) MIA sense transfected melanoma cells (54 ± 3% of visible cells) completed the process of extravasation as compared to the vector control (32 ± 3% of visible cells). MIA antisense transfected cells (26 ± 1% of visible cells) in contrast revealed a reduced ability to cross the endothelial lining indicating a specific role for MIA in the extravasation process of melanoma cells in vivo (Figure 6).

### Liver colonization potential of circulating A-mel 3 cells is dependent on MIA expression

Based on these results, we predicted that rapid extravasation of MIA sense cells should parallel a higher metastatic potential of these cells after intraportal injection. To test this hypothesis we compared the ability of variant A-mel 3 clones to form liver metastases in a comparable metastasis assay. MIA sense transfected cells showed significantly more liver metastases in the subcapsular region than MIA neo cells. MIA antisense A-mel 3 were significantly impaired in their ability to form metastases on the liver surface (Figure 8). We therefore conclude that the altered

 © 2000 Cancer Research Campaign extravasation ability of variant cell clones is paralleled and is therefore causal for differences in the metastatic ability of cells.

### DISCUSSION

It is widely accepted that metastasis is not a random, but in fact a highly selective, process favouring the survival and growth of a few pre-existent subpopulations of cells within the parent neoplasm (Fidler, 1990). Identifying the metastatic subpopulation of cells bears high clinical significance, as prognostic assays based on a random sample of primary tumour would be highly inaccurate in case aggressive cells merely represented a small subset of the total number of tumour cells. In a previous study we were able to show that MIA expression was more pronounced in metastatic lesions than in primary tumour specimens. In addition, MIA serum levels positively correlate with the clinical tumor stage (Bosserhoff et al, 1997a, 1998) and therefore determine prognosis of melanoma patients. Thus, MIA may be a marker of metastatic subpopulations, essential for successful metastasis of individual melanoma cells.

Although information generated from in vitro invasion systems or artificial metastasis models in immunodeficent mice may prove useful, it remains unclear as to how such data could be extrapolated to growth, invasion and metastasis of natural tumours. Furthermore, the invasive and metastatic potential of tumour cells depend on both their intrinsic properties and the host environment at the inoculation site (Fidler, 1996, 1990; Killion et al, 1998). Therefore, we have chosen the A-mel 3 hamster melanoma cell line because it enables in the case of subcutaneous implantation studies in a syngene, immunocompetent model with the primary tumours following the ‘typical’ metastasation route of malignant melanoma. This spontaneous metastasis model mimics several aspects of the metastatic process, requiring the tumour cells to leave the site of initial local tumour growth (primary tumour), migrate through the surrounding tissues, enter and then leave the vascular system, and successfully develop foci of growth in a new environment. In a second approach an experimental metastasis model was used to examine in detail the latter part of this process, i.e. extravasation and initiation of post-extravasational growth.

In the current presentation it is shown that expression of MIA in A-mel 3 melanoma cells positively altered the metastatic capacity of these cells in hamsters, thus providing first direct evidence for the causal involvement of MIA in the metastatic sequence of malignant melanoma. Formation of lymph and lung metastases was drastically enhanced by high levels of MIA, converting mere MIA overexpressing cells into highly metastatic cells. After intraportal injection of tumour cells, an approach that bypasses the release of cells from the primary tumour in the circulation, comparable results were obtained and thus indicating the contribution of MIA to various steps in the metastatic cascade.

MIA expression was not associated with an increase in tumorigenicity. Both MIA transfectants, MIA sense and MIA antisense formed primary tumours corresponding to the size of their respective control vector MIA neo. Therefore differences in the degree of spontaneous metastasis as well as experimental metastasis cannot be due to altered cell proliferation. A second line of evidence originates from in vitro data proving that cell doubling time was unaffected when MIA sense, MIA antisense or MIA neo transfected cells were cultured in vitro in the presence of different concentrations of FCS (1 and 10%). In this respect, Apfel and colleagues have demonstrated that exogenous MIA inhibits cell proliferation in vitro by prolongation of the S-phase and G2 arrest (Bogdahn et al, 1989; Weilbach et al, 1990; Apfel et al, 1992). A higher susceptibility to apoptosis due to a lack of MIA expression as a source of inhibited metastasis in vivo was excluded by TUNEL staining of primary tumours.

To determine whether differences in tumour cell invasion are causal for altered metastasis, both MIA transfectants (MIA sense and MIA antisense) and their respective vector control were tested for their ability to penetrate through the basement membrane in Boyden chamber experiments. This ability mimics an important component in the process of tumour cell invasion and metastasis (Stetler Stevenson et al, 1993). Compared with the respective vector control the MIA overexpressing A-mel 3 cells showed a significantly enhanced invasive capacity, underlining the assumption that MIA might be specifically required to cross tissue boundaries. The loss of invasiveness exhibited by MIA antisense cells was less pronounced. This, however, is plausible taking into consideration that tumour cells do have to transverse ECM and cellular layers at multiple stages, and various motile or antiadhesive properties of cells should add to the pronounced effect observed in the metastasis assays within the metastatic process. Of note, MIA antisense transfected cells do not have a complete MIA knockout and some remnant endogenous MIA secretion does occur.

Our finding that all tumours expressing MIA possess invasive capacity and that the invasive cell number tends to be related to the corresponding level of MIA expression underlines the crucial role of the MIA protein for both the transmembrane and transcellular invasiveness. A role that is supported by our own results obtained by intravital microscopy. Initial tumour cell adhesion, arrest and deformability were not affected by MIA expression. However, we observed a high ratio of MIA overexpressing cells successfully completing the process of extravasation within 24 hours. At this time remaining intravasal tumour cells became gradually destroyed by haemodynamic force. Thus, we propose a central functional role of MIA in those metastatic steps that require cell migration and translocation e.g., the extravasation process.

The process of cell migration requires the coordinated activation of both growth factor and adhesion receptor signaling (Stetler Stevenson et al, 1993). It has been hypothesized that the specific interaction between tumour cells and endothelium via adhesion molecules (homing receptors) at the site of extravasation represents a distinct mechanism determining organ-specific metastasis (Fidler, 1996). In this respect, metastatic cells may resemble normal lymphocytes. However, direct evidence for the involvement of homing receptors in metastasis is scarce and several experiments (Morris et al, 1993; Koop et al, 1995) including our own show that tumour cell adhesion was not involved in the mechanism of initial tumour cell arrest. In our models the formation of metastasis was mainly driven by the site of tumour inoculation and could be attributed to routes of blood flow. Therefore MIA does not mediate organ tropism but may well assist the cells to cross endothelial layers.

So far we do not have any profound functional data regarding MIA function at the molecular level. At the moment it is not clear whether MIA acts via an orphan receptor or uses other structures for its action. The latter is more probable in view of our preliminary results with recombinant MIA showing a sustained inhibition of melanoma cell attachment to fibronectin and laminin, by

© 2000 Cancer Research Campaign blocking $\alpha4\beta1$ and $\alpha5\beta1$ integrin structures (Bosserhoff et al, 1998). Consistent with these findings, other studies report an association between expression of integrins and tumour metastasis. In particular, loss or reduced expression of fibronectin-binding $\alpha5\beta1$-integrin has been associated with malignant progression (Schreiner et al, 1991; Stallmach et al, 1992; Hangan et al, 1997). Conceivable, because of its binding capability to integrin structures, MIA when overexpressed alters cellular anchorage. This in turn leads to disruption of cell–cell adhesion or cell–extracellular matrix interactions. Conversely, underproduction of MIA may restore strong cell–cell and cell–matrix interactions resulting in a reduction of invasive and migratory properties of the affected cells. A similar mechanism involving the secretion of a protein modulating cell–matrix contact and invasion of melanoma cells was recently described for SPARC (Sage and Bornstein, 1991; Ledda et al, 1997). Therefore, MIA probably belongs to a group of several anti-adhesive proteins that includes thrombospondin, tenascin, and SPARC, that signal rapid de-adhesion and potentially mediate migration and proliferation during development of metastasis. Characterization of the precise mechanisms by which MIA modulates adhesive structures and enhances invasion are currently under investigation. It will further be extremely important to determine at what exact stage of the transformation process from melanocytes to progressive malignant melanoma MIA expression occurs and how it is regulated.

In summary, we have been able to show that overexpression of MIA in A-mel 3 melanoma cells induces a metastatic phenotype, enhancing spontaneous lymph node, lung and forced liver metastasis without affecting growth at the primary tumour site or cell proliferation. The explanation for the observed effect was found in a dramatic increase in tumour cell invasion in vitro and extravasation in vivo. Our findings raise the exciting possibility that drugs targeted at MIA could be of eminent clinical value by interfering with tumour progression or metastatic disease in malignant melanoma.

### ACKNOWLEDGEMENTS

The authors thank M Cetto, K Becker and U Holzapfel for excellent technical assistance. We are indebted to Carl Zuelke for careful revision of the manuscript. Animal experiments were performed with the permission of the government of Oberpfalz (AZ40/97) and in accordance to the German animal protection legislation. This work was supported in part by grants from the Deutsche Forschungs Gemeinschaft to R Buettner and AK Bosserhoff.

### REFERENCES

Apfel R, Lottspeich F, Hoppe J, Behl C, Durr G and Bogdahn U (1992) Purification and analysis of growth regulating proteins secreted by a human melanoma cell line. *Melanoma Res* **2**: 327–336

Blesch A, Bosserhoff AK, Apfel R, Behl C, Hessdoerfer B, Schmitt A, Jachimczak P, Lottspeich F, Buettner R and Bogdahn U (1994) Cloning of a novel malignant melanoma-derived growth-regulatory protein, MIA. *Cancer Res* **54**: 5695–5701

Bogdahn U, Apfel R, Hahn M, Gerlach M, Behl C, Hoppe J and Martin R (1989) Autocrine tumor cell growth-inhibiting activities from human malignant melanoma. *Cancer Res* **49**: 5358–5363

Bosserhoff AK, Kaufmann M, Kaluza B, Bartke I, Zirngibl H, Hein R, Stolz W and Buettner R (1997a) Melanoma-inhibiting activity, a novel serum marker for progression of malignant melanoma. *Cancer Res* **57**: 3149–3153

Bosserhoff AK, Kondo S, Moser M, Dietz UH, Copeland NG, Gilbert DJ, Jenkins NA, Buettner R and Sandell LJ (1997b) Mouse CD-RAP/MIA gene: structure, chromosomal localization, and expression in cartilage and chondrosarcoma. *Dev Dyn* **208**: 516–525

Bosserhoff AK, Golob M, Buettner R, Landthaler M and Hein R (1998) [MIA (“melanoma inhibitory activity”). Biological functions and clinical relevance in malignant melanoma]. *Hautarzt* **49**: 762–769

Buettner R, Yim SO, Hong YS, Boncinelli E and Tainsky MA (1991) Alteration of homeobox gene expression by N-ras transformation of PA-1 human teratocarcinoma cells. *Mol Cell Biol* **11**: 3573–3583

Dellian M, Richert C, Gamarra F and Goetz AE (1996) Photodynamic eradication of amelanotic melanoma of the hamster with fast acting photosensitizers. *Int J Cancer* **65**: 246–248

Fidler IJ (1990) Critical factors in the biology of human cancer metastasis: twenty-eighth G.H.A. Clowes memorial award lecture. *Cancer Res* **50**: 6130–6138

Fidler IJ (1996) Critical determinants of melanoma metastasis. *J Investig Dermatol Symp Proc* **1**: 203–208

Hangan D, Morris VL, Boeters L, von Ballestrem C, Uniyal S and Chan BM (1997) An epitope on VLA-6 (alpha6beta1) integrin involved in migration but not adhesion is required for extravasation of murine melanoma B16F1 cells in liver. *Cancer Res* **57**: 3812–3817

Jacob K, Bosserhoff AK, Wach F, Knuchel R, Klein EC, Hein R and Buettner R (1995) Characterization of selected strongly and weakly invasive sublines of a primary human melanoma cell line and isolation of subtractive cDNA clones. *Int J Cancer* **60**: 668–675

Killion JJ, Radinsky R and Fidler IJ (1998) Orthotopic models are necessary to predict therapy of transplantable tumors in mice. *Cancer Metastasis Rev* **17**: 279–284

Koehler MR, Bosserhoff A, von Beust G, Bauer A, Blesch A, Buettner R, Schlegel J, Bogdahn U and Schmid M (1996) Assignment of the human melanoma inhibitory activity gene (MIA) to 19q13.32-q13.33 by fluorescence in situ hybridization (FISH). *Genomics* **35**: 265–267

Koop S, MacDonald IC, Luzzi K, Schmidt EE, Morris VL, Grattan M, Khokha R, Chambers AF and Groom AC (1995) Fate of melanoma cells entering the microcirculation: over 80% survive and extravasate. *Cancer Res* **55**: 2520–2523

Kopf AW, Salopek TG, Slade J, Marghoob AA and Bart RS (1995) Techniques of cutaneous examination for the detection of skin cancer. *Cancer* **75**: 684–690

Ledda MF, Adris S, Bravo AI, Kairiyama C, Bover L, Chernajovsky Y, Mordoh J and Podhajcer OL (1997) Suppression of SPARC expression by antisense RNA abrogates the tumorigenicity of human melanoma cells. *Nat Med* **3**: 171–176

Morris VL, MacDonald IC, Koop S, Schmidt EE, Chambers AF and Groom AC (1993) Early interactions of cancer cells with the microvasculature in mouse liver and muscle during hematogenous metastasis: videomicroscopic analysis [see comments]. *Clin Exp Metastasis* **11**: 377–390

Sage EH and Bornstein P (1991) Extracellular proteins that modulate cell-matrix interactions. SPARC, tenascin, and thrombospondin. *J Biol Chem* **266**: 14831–14834

Schreiner C, Fisher M, Hussein S and Juliano RL (1991) Increased tumorigenicity of fibronectin receptor deficient Chinese hamster ovary cell variants. *Cancer Res* **51**: 1738–1740

Stallmach A, von Lampe B, Matthes H, Bornhoft G and Riecken EO (1992) Diminished expression of integrin adhesion molecules on human colonic epithelial cells during the benign to malign tumour transformation. *Gut* **33**: 342–346

Stetler Stevenson WG, Aznavoorian S and Liotta LA (1993) Tumor cell interactions with the extracellular matrix during invasion and metastasis. *Annu Rev Cell Biol* **9**: 541–573

Weilbach FX, Bogdahn U, Poot M, Apfel R, Behl C, Drenkard D, Martin R and Hoehn H (1990) Melanoma-inhibiting activity inhibits cell proliferation by prolongation of the S-phase and arrest of cells in the G2 compartment. *Cancer Res* **50**: 6981–6986

 *© 2000 Cancer Research Campaign*
